# Cocaine Muriate as a Local Anesthetic

**Published:** 1884-12

**Authors:** 


### COCAINE MURIATE AS A LOCAL ANÆSTHETIC.

A DISCOVERY is just announced which promises to revolutionize the practice of ocular surgery. It has been known for some years that a two-per-cent solution of cocaine muriate, applied to the mucous membrane of the pharynx and vocal cords, diminishes in a remarkable manner the sensitiveness of the parts, and larynxologists have been in the habit of employing such a solution to facilitate their examinations.

Taking the hint from this practice, Dr. Keller, a young physician in Vienna, tested the effect of a solution of cocaine upon the eye. His expectations were more than realized. The solution produced complete anæsthesia of the cornea and conjunctival mucous membrane. Dr. Keller communicated his discovery to Dr. Brettauer, of Trieste, who demonstrated the effects of the new anæsthetic before the Ophthalmological Congress, which recently met in Heidelberg.

A two-per-cent solution of the muriate of cocaine was employed in these experiments. Two drops of the solution were placed in the patient's eye. At the end of ten minutes it was found that the sensitiveness of the superficial structures of the eye was considerably diminished. Two drops more of the solution were introduced into the eye, and after an interval of ten minutes the condition of the eye was once more exaamined, when it was found that the cornea and conjunctival mucous membrane could be freely handled without the least feeling of pain. Probes were passed over the surface of the cornea, conjunctiva bulbi, and conjunctiva palpebrarum, the eyeball was seized with fixation forceps and rotated by them, and a spring speculum was introduced into the eye, distending to the utmost lids, and the patient declared that he felt nothing like pain from any of these experiments. Previous to the instillation of the cocaine solution the eye was shown to have the normal degree of sensitiveness, and only the eye into which this solution had been dropped was affected. The solution caused no irritation of any kind, and the observers reported that the pupil remained unaffected. The anæsthetic influence lasted about fifteen minutes, after which the eye resumed its normal condition.

Dr. H. D. Noyes, of New York, witnessed these remarkable demonstrations, and gave an account of them in a letter published in the Medical Record of Oct. 11. The occulists of New York, recognizing at once the importance of such a discovery, began immediately to experiment for themselves, and one week after the publication of Dr. Noyes' letter, reports of clinical observations by three different oculists appeared in the Medical Record, not only confirming the accounts of the Heidelberg observers, but showing that the importance of the discovery had not been overestimated.

